# Visual Working Memory Encoding and Recognition in Good Outcome Aneurysmal Subarachnoid Patients

**DOI:** 10.3389/fneur.2018.00494

**Published:** 2018-06-26

**Authors:** Leodante da Costa, Priyanka P. Shah-Basak, Benjamin T. Dunkley, Andrew D. Robertson, Elizabeth W. Pang

**Affiliations:** ^1^Division of Neurosurgery, Department of Surgery, Sunnybrook Health Sciences Centre, University of Toronto, Toronto, ON, Canada; ^2^Department of Diagnostic Imaging, The Hospital for Sick Children, Toronto, ON, Canada; ^3^Rotman Research Institute, Baycrest Health Centre, Toronto, ON, Canada; ^4^Neurosciences and Mental Health, Sick Kids Research Institute, Toronto, ON, Canada; ^5^Canadian Partnership for Stroke Recovery, Sunnybrook Research Institute, University of Toronto, Toronto, ON, Canada; ^6^Division of Neurology, The Hospital for Sick Children, University of Toronto, Toronto, ON, Canada

**Keywords:** aneurysm, encoding, magnetoencephalography, recognition, subarachnoid hemorrhage, working memory

## Abstract

**Objectives:** Aneurysmal subarachnoid hemorrhage (aSAH) accounts for less than 5% of strokes but is associated with significant morbidity and mortality. Amongst survivors, neurocognitive complaints are common, often despite normal imaging. We used magnetoencephalography (MEG) to investigate neurophysiological function during a visual working memory task in aSAH survivors with good recovery and normal structural imaging.

**Methods:** Patients with aSAH treated with coiling and exhibiting good outcome measured by Glasgow Outcome Scale (GOS) and without related parenchymal structural lesions in post-treatment MRI, were recruited and compared to age- and sex-matched controls. All participants underwent intelligence and cognitive screening, structural MRI, and MEG testing in conjunction with a 1-back visual working memory task. Sensor-level global field power and virtual electrode source analysis of neuronal activity and connectivity in aSAH were assessed.

**Results:** Thirteen patients and 13 matched controls were enrolled (age: 56 ± 11 years, 19 female). The 1-back task was completed with similar accuracy despite a trend for a longer reaction time in aSAH patients (*p* = 0.054). During encoding and recognition phases, aSAH patients showed significantly increased neuronal activation and hyperconnectivity in periventricular areas, specifically the anterior and posterior cingulate gyri.

**Conclusions:** Increased posterior and anterior cingulate gyri neuronal activity is demonstrated in aSAH patients during visual working memory tasks, in the absence of structural lesions. These areas work mainly as a hub to “organize” memory storage and retrieval. Increased activity in these areas might be compensatory due to injury and consequently loss of neuronal response in connected areas in the working memory networks.

## Introduction

Aneurysmal subarachnoid hemorrhage (aSAH) accounts for less than 5% of all strokes (1, 2). However, it affects patients at a younger age than other types of stroke and has very high morbidity and mortality rates (1, 3). The incidence of aSAH has not changed, but medical advances have decreased mortality over the last few decades (2, 4). Currently most survivors make a good recovery (5) by standard outcome measures, e.g., the Glasgow Outcome Scale (GOS).

This clinical condition still presents as a major burden to both individuals and society, with up to 50% of survivors reporting persistent neurocognitive deficits, not returning to the same level of work (6), and reporting a poor quality of life (7). Often, imaging does not show significant structural abnormality despite the neurocognitive deficits. Functional imaging may be able to identify abnormalities in the absence of structural change. We reported previously that MEG was feasible in this patient population (8), and later showed increased activity in frontal lobe regions related to deficits in inhibitory control and mental flexibility, suggesting a common final pathway underlying different cognitive manifestations in these two neurocognitive domains (9).

Memory deficits are another well-documented neurocognitive complaint in aSAH survivors, even though the precise nature of the memory domains involved and the underlying cause are yet to be determined (10). The aim of this study was to investigate visual working memory function, particularly the short-term maintenance and retrieval aspects of memory (encoding and recognition), in patients with ruptured intracranial aneurysms using MEG. We hypothesized aSAH patients would exhibit poorer performance and altered brain activation measured via MEG compared to healthy matched controls, despite normal structural imaging.

## Materials and methods

### Participants

Patients with ruptured intracranial aneurysms, admitted in good grade and considered to have good GOS-based outcome in follow-up, were recruited. Other inclusion criteria were age ≥18 years; single aSAH with the causative aneurysm treated with uncomplicated coiling; no evidence of ischemic lesion in follow-up imaging; no parenchymal hemorrhage. Acute hydrocephalus and uncomplicated EVD placement were not criteria for exclusion, since it may not correlate with long-term cognitive outcomes (11). Control participants were recruited from the community. Exclusion criteria for both groups were contraindication for MRI scanning, history of previous stroke, neurological or psychiatric disorders, or previous brain surgery. Research Ethics approval was obtained at both institutions (Sunnybrook Health Sciences Centre and The Hospital for Sick Children, Toronto). All participants provided signed informed consent.

### Cognitive assessments

**Figure 1:**
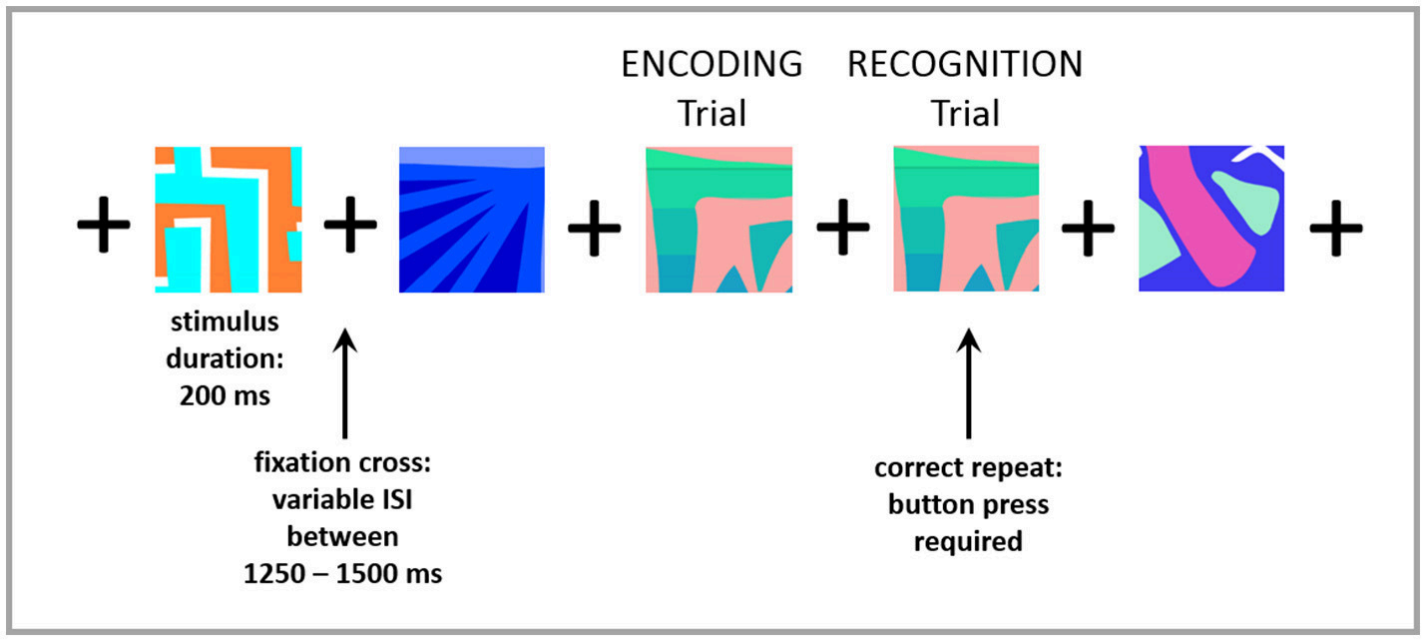
Example of 1-back memory task.

Both groups underwent brief neuropsychological testing to assess full-scale intelligence [Wechsler Abbreviated Scale of Intelligence — WASI (12)] and cognitive function [Montreal Cognitive Assessment — MoCA (13)]. Visual working memory (encoding and recognition) was tested during the MEG session (as described below). Prior to entering the MEG suite, participants were trained on an N-back memory task, modified for MEG (14). The n-back tasks are well established for testing working memory processing in research environments, which typically involve maintenance and manipulation of information (15, 16). In this task, subjects were asked to indicate, by button press, every time that a picture matched the one shown in the immediately previous trial (1-back). Correct matches were labeled as “recognition” trials, and the immediately preceding trial was labeled as “encoding.” Each picture appeared on the screen for 200 ms and was followed by a fixation cross which was presented with a variable inter-stimulus interval of 1,250–1,500 ms, to prevent anticipation of the next trial. Ninety-four repeated stimuli (“recognition trials”) were presented. See Figure 1 for an example of the task. Accuracy and reaction time (RT) were recorded and compared between groups. Accuracy was measured as the ratio of the number of correctly recognized trials to the total number of trials.

### MEG and MRI acquisition

MEG data were acquired with participants in a supine position, using a 151-channel whole-head MEG system (CTF Omega, MISL Inc., Coquitlam, Canada) at 600 Hz with a 200 Hz low-pass filter and third-order spatial gradient noise cancelation. Fiducial coils were placed on the nasion and bilateral pre-auricular points to monitor head position. The MEG trials corresponding to correct *recognition* of the repeated stimuli and the preceding *encoding* trials were analyzed and compared between-groups. After completion of the MEG recording, a structural MRI was acquired using a 3T scanner with a 12-channel head coil (Magnetom Tim Trio, Siemens AG, Erlangen, Germany). The MRI was a T1-weighted sagittal scan using an MPRAGE sequence (repetition time = 2,300 ms; echo time = 2.96 ms; flip angle = 9°; field-of-view = 192 × 240 × 256, 1mm istropic resolution). Localization coils placed during the MRI allowed data to be co-registered with the MEG reference fiducial coils.

### MEG and MRI processing (see Supplementary Material)

Continuous MEG data were band-pass filtered offline at 1–50 Hz for evoked analysis and 1–150 Hz for connectivity. Sensor-level data for *encoding* and *recognition* trials were inspected by computing global field power (GFP) on time-locked averages across participants. Cortical and sub-cortical sources of interest were identified as 90 pre-defined seed locations from the Automated Anatomical Labelling Atlas (AAL) (17) and used for source power and connectivity analyses. Time-series data were reconstructed from these locations using a vector beamformer (linearly constrained minimum variance) for each participant.

For functional connectivity analyses, source-level time series were recovered from 90 AAL locations, filtered into 5 canonical bandwidths (Theta [4–7 Hz], Alpha [8–14 Hz], Beta [15–30 Hz] and Gamma [30–55 Hz]), and submitted to functional connectivity analysis using the Phase Lag Index (PLI) (18). For the final analysis, we have focused on the connectivity findings in the theta band because of its long-standing and widely accepted role in working/short-term and recognition memory processing (19–23). Structural analysis was carried out with freely available software, Freesurfer 5.1 (http://surfer.nmr.mgh.harvard.edu).

### Statistical analysis

Task performance and cognitive assessment scores between groups were compared using non-parametric Mann-Whitney-Wilcoxon test for continuous variables and Fisher's exact tests for categorical variables. Statistical significance was inferred at *p* < 0.05 and statistical trends were inferred at *p* < 0.1. To control for differences in IQ between groups for the assessment of task performance, linear regression analysis was conducted (Table 1). Furthermore, Spearman's partial correlations were conducted to assess associations between study variables and task performance after controlling for IQ within the patient group. RStudio (version 0.98.945) was used for all behavioral analyses.

**Table 1:** Group demographics and aneurysm location, time to MEG and cognitive scores and task results.

	**Control**	**aSAH**	***p*-value**
*N*	13	13	
Age (years)	56.7 (±10.5)	55.7 (±10.0)	n.s.
Sex	9F; 4M	8F; 5M	^†^n.s.
Education (years post-secondary)	1.92 (±0.083)	1.54 (±0.14)	0.03
Handedness	12R; 1L	11R; 2L	^†^n.s.
MoCA	24.9 (±3.2)	24.9 (±3.4)	n.s.
WASI	120.4 (±14.7)	103.8 (±15.4)	0.01
**ANEURYSM LOCATION**			
AComm		6	
PComm		3	
PICA		1	
SCA		1	
ACA		1	
Basilar		1	
**Time from aSAH to MEG (months)**			12.5 (±9.6)
**1-BACK MEMORY TASK**			
Accuracy (%)	84.5 (±17.1)	87.9 (±12.2)	n.s.
Reaction time (ms)	470.2 (±102.5)	516.5 (±74.6)	0.056

Data are presented as means (±standard deviations) or counts. aSAH, Aneurysmal subarachnoid hemorrhage; MoCA, Montreal Cognitive Assessment; WASI, Wechsler Abbreviated Scale of Intelligence; Fisher's Exact Test.

*AComm, anterior communicating artery; PComm, posterior communicating artery; PICA, posterior inferior cerebellar artery; SCA, superior cerebellar artery; ACA, anterior cerebral artery*.

Source power estimations were subjected to non-parametric statistical testing using the Monte Carlo method with 1,000 randomizations and T-statistic for between-group comparisons. False discovery rate (FDR) correction was applied to correct for multiple comparisons and significant maps were generated at p_FDR−corrected_ < 0.05. FieldTrip functions (24) were used for these analyses. The source activity was extracted from the local maxima within significant sources to visualize the differences in evoked power.

For functional connectivity analyses, non-parametric permutation testing using Network Based Statistic (NBS) Toolbox (25) was used to investigate within-group differences in connectivity during encoding and recognition trials as compared with the baseline (NBS: Intensity method; initial suprathreshold *t*-value = 2, *p* < 0.05, 10,000 permutations). Graph analysis was performed using the Brain Connectivity Toolbox (BCT) (26). *Node strength*, the sum of edge weights connecting a node to the rest of the network, was computed using BCT to quantify the local properties of the network. Binary brain networks representing significant within-group connectivity differences were visualized using BrainNet Viewer (27) with the nodal strength measure. Lastly, the subcortical structure volumes were compared between groups using *t*-tests with Bonferroni correction (*p*_Bonferroni−corrected_ < 0.05).

## Results

Sample demographics, as well as neuropsychological performance, aneurysm location and time from aSAH to MEG testing (study intervals), are contained in Table 1. Mean age, sex distribution and right-handedness were similar between patients and controls. Controls had significantly more years of post-secondary education and higher WASI (IQ) scores (effect size = 0.51); however, there was no difference in the MoCA total score. The 1-back task accuracy was similar between groups (overall mean accuracy: 85%), however aSAH patients tended to require more time (slower by 46.3 ms; *p* = 0.056; effect size = 0.38). After controlling for IQ scores, the reaction time differences between groups remained marginally significant (β = 6.3, *t* = 2.24; *p* = 0.055). The differences in accuracy were not significant. In the patient group, the study interval was not correlated with RT or accuracy (*p* > 0.05) after controlling for IQ. This suggests that in our patient cohort, chronicity of aSAH or IQ did not affect the behavioral performance.

**Figure 2:**
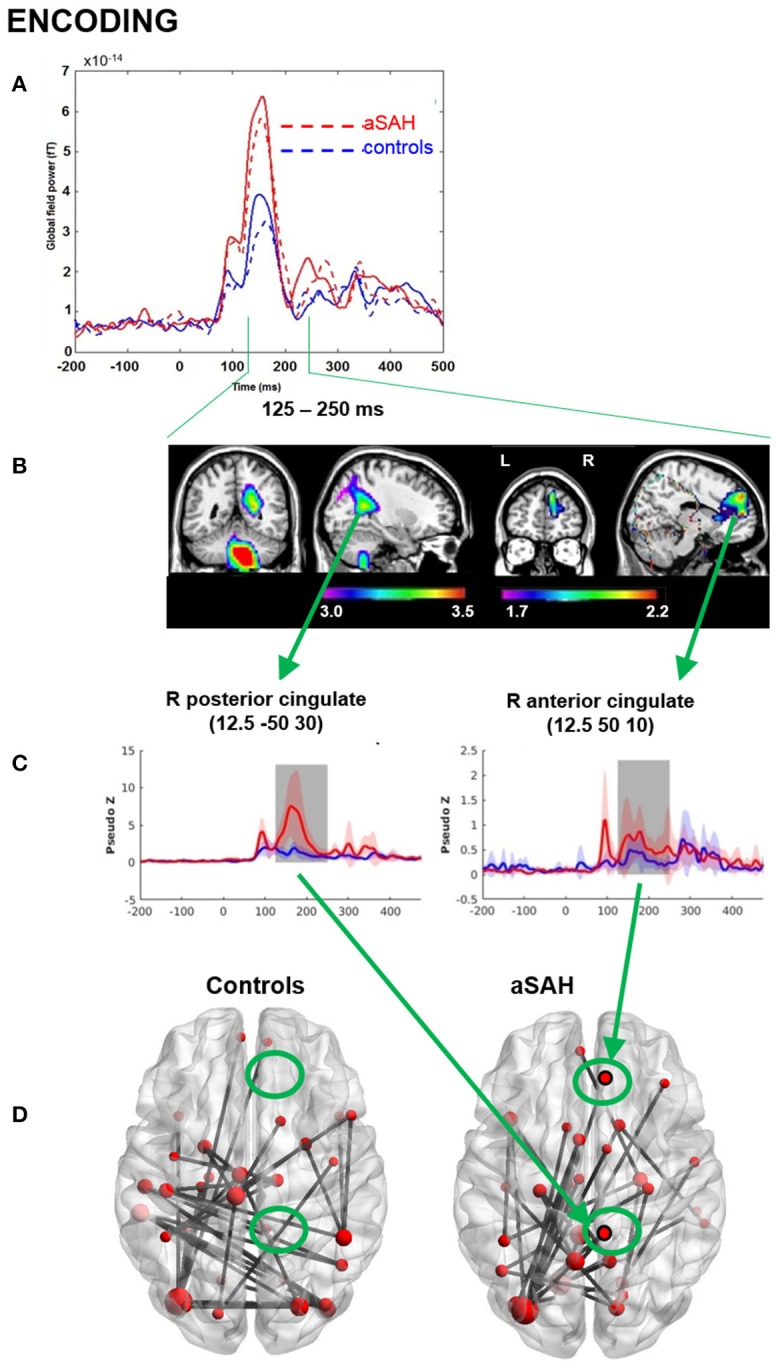
MEG results from memory encoding. **(A)** Global field power plot from all sensors showing between group and between task differences. Encoding: dotted line. **(B)** Beamformer source localization of group difference aSAH > controls (*P* < 0.05_*corr*_). **(C)** Reconstructed time course at cingulate locations aSAH > controls. **(D)** Cingulate shows significant connectivity in aSAH but not in controls intensity, theta band, 125-250 ms, *p* < 0.05, nPerm = 10,000.

**Figure 3:**
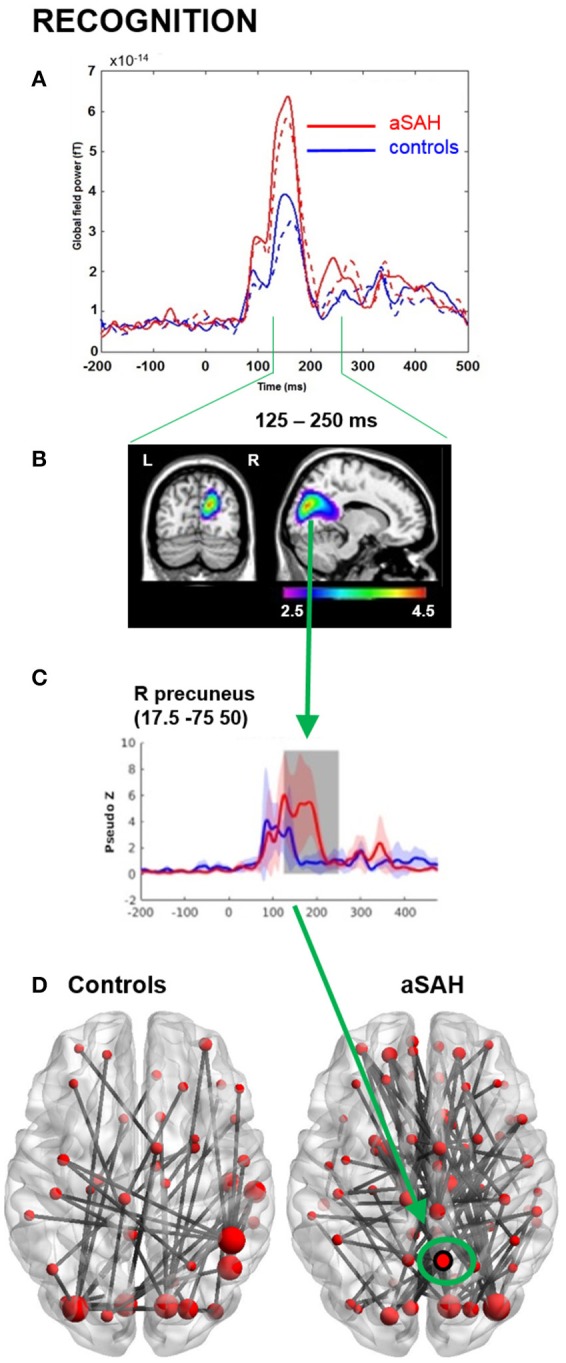
MEG results from memory recognition. **(A)** Global field power plot from all sensors showing between group and between task differences. Recognition: solid line. **(B)** Beamformer source localization of group difference aSAH > controls (*P* < 0.05_*corr*_). **(C)** Reconstructed time course at precuneus locations aSAH > controls. **(D)** Over-connectivity in aSAH, particularly in right precuneus intensity, theta band, 125-250 ms, *p* < 0.05, nPerm = 10,000.

Neurophysiological visual working memory function was examined during encoding and recognition phases. Visual inspection of the GFP plots indicated that aSAH patients displayed much higher evoked functional activation during both encoding (Figure 2A) and recognition (Figure 3A), particularly in the 125–250 ms time window for the former, extending into the 250–375 ms window for the latter.

Consistent with GFP, evoked source analyses identified higher neuronal activation in aSAH than controls in certain regions (Table 2; Figures 2B,C, 3B,C) during both encoding and recognition. Overall, our findings show much greater involvement of right medial parietal and cingulate areas in aSAH patients during this visual working memory task.

**Table 2:** MEG sources of significant activation differences between groups during 3 time windows of interest (0–125 ms; 125–250 ms; 250–375 ms) in Encoding and Recognition trials.

**Time windows**	**Group differences**	**Brain area**		**MNI coordinates**
**ENCODING**				
0–125 ms	aSAH > Control	L	Inferior Temporal	[−57.5, −40, −15]
125–250ms	aSAH > Control	R	Cuneus	[17.5, −30, −5]
		R	Precuneus	
		R	Posterior Cingulate	[12.5, −50, 30]
		R	Anterior Cingulate	[12.5, 50, 10]
250–375 ms	aSAH > Control	R	Precuneus	[17.5, −75, 50]
		R	Superior Occipital	
		R	Superior Parietal	
		R	Middle Temporal	[52.5, −45, 0]
**RECOGNITION**				
0–125 ms	aSAH > Control	L	Inferior Temporal	[−57.5, −45, −20]
125–250 ms	aSAH > Control	R	Cuneus	
		R	Precuneus	
		R	Superior Occipital	
		R	Calcarine	

Connectivity findings were congruent with evoked findings, and suggested heightened involvement of the right precuneus/cuneus and cingulate areas in the theta band during encoding and recognition (Figures 2D, 3D), with a visually noticeable over-connectivity for the aSAH group during recognition (Figure 3D).

Structural analysis revealed smaller volumes in the nucleus accumbens in aSAH compared to controls (*p*_Bonferroni−corrected_ = 0.048). Uncorrected *p*-values also suggested trends for smaller volumes in the amygdala, caudate nucleus, hippocampus, and thalamus in aSAH (Table 3).

**Table 3:** Normalized subcortical brain volumes.

	**Control**	**aSAH**	**Uncorrected *p*-value**
ICV, mL	1331 ± 159	1425 ± 186	0.117
Accumbens, %	0.05 ± 0.01	0.04 ± 0.01	0.007
Amygdala, %	0.12 ± 0.01	0.11 ± 0.02	0.009
Caudate, %	0.26 ± 0.03	0.23 ± 0.04	0.033
Hippocampus, %	0.31 ± 0.03	0.28 ± 0.05	0.038
Pallidum, %	0.12 ± 0.01	0.11 ± 0.02	0.064
Putamen, %	0.39 ± 0.05	0.35 ± 0.06	0.059
Thalamus, %	0.48 ± 0.06	0.44 ± 0.06	0.039

*ICV, intracranial volume. Mean ± SD*.

## Discussion

The main findings of our study are the differences in neuronal activation during the execution of tasks designed to probe visual working memory encoding and recognition between aSAH survivors with good outcomes and controls. Increased activity in aSAH was noted mostly in periventricular regions and parieto-occipital lobes, an anatomical distribution in line with the previously identified networks that are activated during working memory tasks (14). Despite the increased activity, the findings do not support part of our initial hypothesis that aSAH patients' performance would be worse than controls. The posterior and the anterior cingulate gyrus (pre-frontal cortex) are hyperactive in aSAH patients during the memory tasks. Interestingly, anterior cingulate cortex hyperactivity was also demonstrated during inhibition and mental flexibility tasks in a previous report (9), suggesting a common pathway, or at least a prominent role of these regions in neurocognitive deficits after injury caused by a ruptured brain aneurysm.

Aneurysmal SAH continues to be a cause of significant morbidity and mortality despite significant advances in care (28). Because it affects a younger population compared to other types of stroke (28), the long-term personal and socioeconomic burden is significant (6, 29). Cognitive deficits and depression are common after aSAH (28, 30), with memory deficits identified in approximately 60% of survivors (28, 31). Neurocognitive deficits can persist beyond 1 year (5, 31, 32) and, more importantly, these deficits have stronger correlation with poor Health-Related Quality of Life scores than physical impairments (33). Despite its prevalence and significance, the specific pathophysiological mechanisms behind the neurocognitive complaints in patients without identifiable anatomical injuries are yet to be determined.

It has been shown that neurocognitive deficits are correlated to the severity of initial presentation and volume of subarachnoid blood (5, 11). These however are well known markers of overall poor outcome after aSAH, and might not be specific markers for neurocognitive/memory problems in patients with “good recovery.” The role of other complications of aSAH such as hydrocephalus, vasospasm and associated ischemic complications in the development of neurocognitive deficits in this population is less clear (11, 31, 34).

The inclusion criteria in our study only allowed enrollment of patients with recent MRI without any evidence of structural brain damage. Neurophysiological data (MEG) collected in this study show that long-lasting or permanent neuronal dysfunction may be present in the absence of neuroanatomical injury and can be detected with current functional imaging technology, as demonstrated previously in humans (9) and animal models (35). It seems that a condition of “neuronal inefficiency” is created by the injury, based on animal work and previous studies in mild traumatic brain injury (36, 37) and aSAH (9).

The trend toward longer times required to complete the same task by aSAH patients is another indication of inefficient neuronal activation and/or networking. Using functional magnetic resonance imaging (fMRI), van Snellenberg et al. investigated network activation in working memory tasks (38). By increasing task difficulty progressively, they demonstrated an inverted U pattern of network activation, with eventual decrease in recruitment as tasks became more difficult. Individuals with more working memory capacity failed at later stages. Our findings support the hypothesis of “reduced capacity” for working memory, reflected in early recruitment of additional brain networks to continue to cope with task requirements.

In the patient group, the time to MEG was not correlated with reaction time or the accuracy after controlling for IQ. This suggests that in our patient cohort, chronicity of aSAH or IQ did not affect their behavioral performance. We speculate that while these important variables were not significantly affected on this relatively less cognitively demanding task (i.e., 1-back), they may significantly influence patient performance on more intensive tasks, such as the 2-back. This question remains to be addressed in future studies.

The processes through which new memories are formed and stored are complex and not completely understood. The formation of new memories requires the creation of a new memory network. This process was thought to happen in the hippocampus, and the new memory would be later transferred to the cortex for storage. More recently, it has been suggested that new memories are initially encoded in the hippocampus, and later assimilated into existing memory networks in the neocortex (39). The prefrontal cortex would then play an important role in the organization of neocortical representations of these memories and, later on, retrieval.

In a rat model of SAH, Tariq et al. showed that long-term hippocampus potentiation is disrupted following aSAH (40). This process is thought to be the molecular basis of memory and a fundamental step in learning and memory. Furthermore, they observed little neuronal damage and no evidence of cell death, suggesting that the disruption is not the result of apoptosis but rather functional impairment. In a similar model, Han et al. showed the number of synapses in the hippocampus is reduced and the expression of key proteins involved in long term potentiation is decreased in CA1 (41). These observations were in the absence of ischemic lesions, neuronal death, decreased cerebral blood flow or changes in glucose metabolism, further supporting the idea of a functional change without visible lesion in current imaging techniques.

It has been shown that some SAH patients with prefrontal cortex damage do not have severe impairments in standard tests of event memory, but deficits are apparent when memory related to specific information must be obtained under conditions of memory interference (42). For example, individuals with prefrontal damage demonstrate impaired performance on the classic A–B, A–C problem (42). After learning a set of paired associations (e.g., A-B) patients with prefrontal cortex injuries have difficulty in learning new associations (e.g., A-C). These patients often also have issues memorizing one of two lists of unrelated associations due to intrusions of items from one list in the other.

Functions of the prefrontal cortex and anterior cingulate gyrus include modulation of complex neuronal activity related to cognition and memory control. As far as memory is concerned, one of the theories able to explain the relationship between the prefrontal cortex and the hippocampus can be illustrated with the “railroad metaphor.” The hippocampus would be responsible for laying down the new “memory tracks,” and the prefrontal cortex would coordinate their use and switch tracks as required for efficient use of resources and conflict avoidance (42).

Based on this theory, one can speculate that increased activity detected in the prefrontal cortex can be related to compensatory activity due to injury and consequently loss of neuronal response from either the prefrontal cortex itself and / or the hippocampus. This is obviously a simplistic approach to such a complex issue, but there is evidence that the role of the prefrontal cortex in memory is one of organizational and strategic control of the memory retrieval processes (42). Also, we cannot rule out the hypothesis that this finding is due simply to “up-regulation” of the region after the aSAH for an unexplained reason. This idea of neuronal “inefficiency” seems to be similar to the one seen with mild traumatic brain injury, where a significantly larger number of brain regions are activated to achieve similar task performance as compared to healthy controls (36, 37, 43). Unfortunately, we were not able to detect which region(s) in the brain is (are) dysfunctional to support this theory. Although the idea that the hyperactivity detected is consequence of loss of neuronal efficiency and therefore more connections and activity is required to maintain performance, we recognize that this work does not prove such assumption.

Interestingly, we also found increased neuronal activity in the posterior cingulate cortex, and increased connectivity between the anterior and posterior frontal cortices. The posterior cingulate cortex is a large area in the posterior aspect of the corpus callosum with established roles in the default mode network, internally directed cognition and attention focus. The region is characterized by very high metabolism rates, and although it is often treated as a unit, significant functional and anatomical heterogeneity within the PCC has been demonstrated (44, 45).

The PCC role in memory is better established for autobiographical or emotionally loaded memories (44, 46). The posterior cingulate gyrus is a “task negative” region, being *deactivated* during task execution (47), which is in alignment with the region's connections with the default mode network (DMN). Through its participation in the DMN, the PCC seems to be instrumental in regulation of awareness. Lack of deactivation is correlated with poorer performance on memory related tasks (44), likely due to excessive “interference” during memory retrieval. Similar to the anterior cingulate and prefrontal cortex, the PCC seems to play a major role in multiple domains, being connected to multiple brain networks. Increased activity in this area during memory tasks can also be related to lack of proper feedback from other memory network regions. Due to the negative correlation between PCC activity and memory performance demonstrated previously (44), however, up-regulation and increased activity in this region might also lead to delays in tasks. An alternative hypothesis would be that the PCC is up regulated, and its increased activity interferes with function in other memory areas.

Although no notable anatomical injuries were present, the volume of key subcortical structures tended to be smaller in aSAH (Table 3). Case-control differences have previously been reported in the hippocampus and amygdala (48). We observed the largest difference in the nucleus accumbens, which may have greater implications for associative tasks (49). Although such tasks were not considered in the current paradigm, they can greatly impact quality of life for aSAH patients. The generalized subcortical atrophy that we observed may particularly impact the limbic system, including functional networks between the hippocampus, amygdala and anterior cingulate (50). Hippocampal atrophy in particular supports the hypothesis that increased anterior cingulate activation may be an attempt to compensate for subtle impairments in memory encoding, as discussed above.

Our study has a small sample, and patients were strictly selected in order to avoid confounding factors (e.g., craniotomy, intraparenchymal bleed). Therefore, the results in this population may not apply to ruptured aneurysms in general, such as patients with parenchymal lesions (ischemic and/or hemorrhage), vasospasm, or patients submitted to microsurgical clipping. Also, the WASI results at baseline were different between groups, which we assume is due to bias in recruiting controls with longer post-secondary education.

## Conclusion

This study shows that despite similar performance, aSAH survivors have significantly increased neuronal activity in periventricular areas of the brain related to working memory and neurocognitive control, specifically the anterior and posterior cingulate regions. This was found in patients with good outcomes by standard measures and without structural brain injury related to the previous aneurysm rupture and treatment. We speculate that these differences in neuronal activation may be related to a state of neuronal innefficiency in memory networks connected to the posterior and anterior cingulate cortices caused by the aSAH and might explain the subtle and often persistent cognitive issues that impact these patients. Future analyses of memory networks using local and global graph theory measures would further elucidate the sources of neuronal inefficiency in aSAH.

## Ethics statements

This study was carried out in accordance with the recommendations of Tri-Council Policy Statement - Ethical Conduct for Research Involving Humans, Canadian Institutes of Health Research, Natural Sciences and Engineering Research Council of Canada and Social Sciences and Humanities research Council of Canada. The protocol was approved by the Research Ethics Board at Sunnybrook Health Sciences Centre and at the Hospital for Sick Children, Toronto, Ontario. All subjects gave written informed consent in accordance with the Declaration of Helsinki.

## Author contributions

LdC conceived and helped to design the study, secured funding, helped with patient recruitment and wrote the manuscript draft. PS-B and BD analyzed the MEG data and provided results and structural maps. AR analyzed volumetric structural imaging. All authors contributed reviewing the manuscript draft in preparation for its final form. LdC and EP approved the final version.

### Conflict of interest statement

The authors declare that the research was conducted in the absence of any commercial or financial relationships that could be construed as a potential conflict of interest.
