# Supplementary material for: Visual Working Memory Encoding and Recognition in Good Outcome Aneurysmal Subarachnoid Patients
Source: Front Neurol. 2018 Jun 26;9:494. doi: 10.3389/fneur.2018.00494 (PMC6028596; doi:10.3389/fneur.2018.00494)
Supplement: Supplementary file 1 [file Data_Sheet_1.DOCX]

*MEG and MRI Processing*

After segmentation of the brain surface, single shell approximation method^1^ was used to construct a volume conduction model. Isotropic grids (5mm) were constructed as source models in each participant. The continuous MEG data were band-pass filtered offline at 1-50 Hz for evoked analysis and 1-150Hz for connectivity using a 5^th^ order filter; a notch filter (60Hz powerline frequency, 8 Hz bandwidth) was also applied for the latter. Sensor-level data for *encoding* and *recognition* trials were inspected by computing global field power (GFP) on time-locked averages across participants. Source-level analyses were based on GFP peaks observed in 3 non-overlapping time windows: 0-125 milliseconds (ms), 125-250ms and 250-375ms.

Cortical and sub-cortical sources of interest were identified using the 5mm isotropic grid for evoked and 90 pre-defined seed locations using Automated Anatomical Labelling Atlas (AAL)^2^ for connectivity. Time-series data were reconstructed from these locations using a vector beamformer (linearly constrained minimum variance) for each participant. Beamforming is an adaptive spatial filtering technique that allows for accurate source-level activity reconstruction and artefact suppression from sources not of interest (e.g., physiological and environmental artefacts)^3^. To recover virtual electrode time series, sensor-level MEG data are projected through individual weight vectors and summated to give estimated source activity at a particular brain location^4, 5^. Power was computed within the time windows of interest at each grid point and was subjected to nonparametric statistical test using Monte Carlo estimate on 1000 randomizations and T-statistic for between-group comparisons. False discovery rate (FDR) correction was applied to correct for multiple comparisons and significant maps were generated at p_FDR-corrected_ < 0.05. FieldTrip functions^6^ were used for these analyses. The source activity was extracted from the local maxima within significant sources to visualize the differences in evoked power.

For functional connectivity analyses, source-level time series were recovered from 90 AAL locations, filtered into 5 canonical bandwidths (Theta [4-7Hz], Alpha [8-14Hz], Beta [15-30Hz] and Gamma [30-55Hz]), and submitted to functional connectivity analysis using the Phase Lag Index (PLI)^7^. The instantaneous phase of each sample from the band-limited time-series was derived from a Hilbert transform. The PLI describes the degree of phase synchronization between all pairwise seed combinations in the source model^8^. Varying between 0 and 1, it quantifies the consistency of the phase angle difference between two seed/sources (seed-seed connectivity) at each sample, across all trials (inter-seed cross-trial phase synchrony). Non-parametric permutation testing using Network Based Statistic (NBS) Toolbox^9^ was used to investigate within-group differences in connectivity during encoding and recognition trials as compared with the baseline (NBS: Intensity method; initial suprathreshold t-value=2, p<0.05, 10,000 permutations). Graph analysis was performed using the Brain Connectivity Toolbox (BCT)^10^. *Node strength*, the sum of edge weights connecting a node to the rest of the network, was computed using BCT. Brain networks were visualized using BrainNet Viewer^11^.

Structural analysis was carried out with freely available software, Freesurfer 5.1 (<http://surfer.nmr.mgh.harvard.edu>). T1-weighted images underwent skull stripping, B1 bias field correction, intensity normalization, registration to Talairach space and volumetric subcortical segmentation^12-14^. Manual corrections for obvious deviations in skull stripping were performed. Subcortical structure volumes were averaged between the right and left hemispheres, normalized by intracranial volume to account for differences in head size^15^, and compared between groups.

References

1. Nolte G. The magnetic lead field theorem in the quasi-static approximation and its use for magnetoencephalography forward calculation in realistic volume conductors. *Physics in medicine and biology*. 2003;48:3637

2. Tzourio-Mazoyer N, Landeau B, Papathanassiou D, Crivello F, Etard O, Delcroix N, et al. Automated anatomical labeling of activations in spm using a macroscopic anatomical parcellation of the mni mri single-subject brain. *NeuroImage*. 2002;15:273-289

3. Muthukumaraswamy SD. High-frequency brain activity and muscle artifacts in meg/eeg: A review and recommendations. *Frontiers in human neuroscience*. 2013;7:138

4. Dunkley BT, Doesburg SM, Sedge PA, Grodecki RJ, Shek PN, Pang EW, et al. Resting-state hippocampal connectivity correlates with symptom severity in post-traumatic stress disorder. *Neuroimage Clin*. 2014;5:377-384

5. Dunkley BT, Da Costa L, Bethune A, Jetly R, Pang EW, Taylor MJ, et al. Low-frequency connectivity is associated with mild traumatic brain injury. *Neuroimage Clin*. 2015;7:611-621

6. Oostenveld R, Fries P, Maris E, Schoffelen JM. Fieldtrip: Open source software for advanced analysis of meg, eeg, and invasive electrophysiological data. *Comput Intell Neurosci*. 2011;2011:156869

7. Vinck M, Oostenveld R, van Wingerden M, Battaglia F, Pennartz CMA. An improved index of phase-synchronization for electrophysiological data in the presence of volume-conduction, noise and sample-size bias. *NeuroImage*. 2011;55:1548-1565

8. Lau TM, Gwin JT, McDowell KG, Ferris DP. Weighted phase lag index stability as an artifact resistant measure to detect cognitive eeg activity during locomotion. *J. Neuroeng. Rehabil*. 2012;9:1-9

9. Zalesky A, Fornito A, Bullmore ET. Network-based statistic: Identifying differences in brain networks. *NeuroImage*. 2010;53:1197-1207

10. Rubinov M, Sporns O. Complex network measures of brain connectivity: Uses and interpretations. *NeuroImage*. 2010;52:1059-1069

11. Xia M, Wang J, He Y. Brainnet viewer: A network visualization tool for human brain connectomics. *PloS one*. 2013;8:e68910

12. Fischl B, Salat DH, van der Kouwe AJ, Makris N, Segonne F, Quinn BT, et al. Sequence-independent segmentation of magnetic resonance images. *NeuroImage*. 2004;23 Suppl 1:S69-84

13. Fischl B, Salat DH, Busa E, Albert M, Dieterich M, Haselgrove C, et al. Whole brain segmentation: Automated labeling of neuroanatomical structures in the human brain. *Neuron*. 2002;33:341-355

14. Dale AM, Fischl B, Sereno MI. Cortical surface-based analysis. I. Segmentation and surface reconstruction. *NeuroImage*. 1999;9:179-194

15. Buckner RL, Head D, Parker J, Fotenos AF, Marcus D, Morris JC, et al. A unified approach for morphometric and functional data analysis in young, old, and demented adults using automated atlas-based head size normalization: Reliability and validation against manual measurement of total intracranial volume. *NeuroImage*. 2004;23:724-738
